# Initial Experience with Proton Beam Therapy for Differentiated Thyroid Cancer

**DOI:** 10.14338/IJPT-D-20-00053

**Published:** 2021-06-25

**Authors:** Nathan Y. Yu, Aditya Khurana, Daniel J. Ma, Michelle A. Neben-Wittich, Michael A. Golafshar, Lisa A. McGee, Jean-Claude M. Rwigema, Robert L. Foote, Samir H. Patel

**Affiliations:** 1 Department of Radiation Oncology, Mayo Clinic, Phoenix, AZ, USA; 2 Mayo Clinic Alix School of Medicine, Mayo Clinic, Scottsdale, AZ, USA; 3 Department of Radiation Oncology, Mayo Clinic, Rochester, MN, USA; 4 Division of Health Sciences Research, Mayo Clinic, Scottsdale, AZ, USA

**Keywords:** thyroid cancer, proton beam therapy, intensity-modulated proton therapy

## Abstract

**Purpose:**

External beam radiotherapy is used in a subset of high-risk patients with differentiated thyroid cancer (DTC). Recurrent, radioactive iodine (RAI)–refractory DTC carries a poor prognosis. We report our initial experience of intensity-modulated proton therapy (IMPT) for recurrent, RAI-refractory DTC.

**Patients and Methods:**

Fourteen patients with recurrent, RAI-refractory DTC were consecutively treated with IMPT from November 2016 to March 2020 at our multisite institution. Patient, tumor, and treatment characteristics were recorded. Overall survival and local-regional recurrence-free survival were recorded and estimated using the Kaplan-Meier method. Acute and late treatment-related toxicities were recorded based on the Common Terminology Criteria for Adverse Events version 5.0. Patients completed the European Organization for Research and Treatment of Cancer Quality of Life Head and Neck Module at baseline and after IMPT. Eleven patients were included in the final analysis.

**Results:**

Median follow-up was 8 months (range, 3-40) for all patients. Median age at treatment with IMPT was 64 years (range, 40-77), and the majority were men (64%). Recurrent histologies included papillary (55%), Hurthle cell (36%), and poorly differentiated (9%) carcinoma; 1 patient had tall cell variant. Concurrent chemotherapy was not administered for any patient in this cohort. At 8 months, all patients were alive without local-regional failure. Acute grade 3 toxicities were limited to 1 patient with dysphagia, requiring feeding tube placement. Two patients experienced late grade 3 esophageal stenosis requiring dilation. There were no grade 4 or 5 toxicities. There were no differences in pretreatment versus posttreatment patient-reported outcomes in terms of dysphagia or hoarseness.

**Conclusion:**

In our early experience, IMPT provided promising local-regional control for recurrent, RAI-refractory DTC. Further study is warranted to evaluate the long-term efficacy and safety of IMPT in this patient population.

## Introduction

Researchers projected that approximately 53,000 new cases of thyroid cancer would occur in the United States in 2020 and that the incidence is increasing 3% per year [1, 2]. Differentiated thyroid cancer (DTC) accounts for the vast majority of thyroid cancer. Standard-of-care treatment for de novo DTC is surgical resection with adjuvant radioactive iodine (RAI) therapy (I-131) in selected patients and thyroid hormone suppression therapy. The role of external beam radiotherapy (EBRT) in DTC is controversial [3–15]. There are few effective treatments for locally or regionally recurrent, RAI-refractory DTC. Most patients with recurrent DTC undergo salvage surgical resection with potential additional RAI and percutaneous ethanol ablation [16–19]. EBRT has been used in patients with RAI nonavid or refractory disease, in patients with gross residual or unresectable primary or recurrent disease, and in high-risk patients >45 years old [3–5].

Delivery of tumoricidal doses to gross disease can lead to significant morbidity due to the proximity of critical structures [20]. There is significant interest in improving the therapeutic ratio with novel radiotherapy (RT) technology. Intensity-modulated radiotherapy (IMRT), the most advanced form of photon EBRT, has demonstrated feasibility and efficacy for nonanaplastic thyroid cancer [21]. Proton beam therapy (PBT) has the potential to further improve the therapeutic ratio due to the Bragg peak phenomenon [22]. The most advanced form of PBT, intensity-modulated proton therapy (IMPT), has further dosimetric advantages over both 3-dimensional PBT (3D-PBT) and conventional photon EBRT [22]. Although the relative biological effectiveness (RBE) of PBT is assumed to be 1.1, it is known that the RBE is variable and that more pronounced biological effects are possible, which can be useful when treating indolent histologies such as recurrent DTC [23].

PBT is being used more frequently at multiple institutions for head and neck cancer. The clinical outcomes of PBT for DTC have not been formally reported. Therefore, we report our initial multisite experience of patients with recurrent, radioactive iodine (RAI) refractory DTC treated with IMPT.

## Patients and Methods

This study was approved b the institutional review board. Between November 2016 and March 2020, 14 patients with recurrent, RAI-refractory DTC were treated with definitive IMPT at our multisite institution. Consecutively treated adults ≥18 years old with recurrent, RAI-refractory DTC treated with IMPT with curative intent were included in this study. Eleven patients were included in the final analysis; 1 patient was excluded due to lack of posttreatment follow-up, and 2 patients were excluded due to hypofractionated treatment. No patients received EBRT to the head and neck region before treatment with IMPT.

At the time of recurrence, a history and physical exam were completed for all patients by a head and neck surgeon, medical oncologist, and radiation oncologist. Staging computed tomography (CT) and magnetic resonance imaging or positron emission tomography (PET) imaging were obtained for all patients. The majority of patients underwent CT of the neck with intravenous contrast. All patients were classified as having recurrent disease based on biopsy (82%) or PET/CT imaging (18%). All patients had detectable thyroglobulin tumor marker before IMPT. All patients had received adjuvant RAI as part of their initial treatment for de novo DTC before presentation for recurrent disease. All patients were reviewed by a multidisciplinary team, and RT treatment and modality were determined based on age, prior treatment, performance status, and tumor location.

All patients were simulated and treated in the supine position with a custom head and neck thermoplastic immobilization device with an intraoral stent. High-resolution CT simulation images (4-dimensional at the discretion of the treating radiation oncologist) were obtained for target delineation and treatment planning purposes. Intravenous contrast was used at the discretion of the treating radiation oncologist. Treatment target volumes were delineated per the respective radiation oncologist. Generally, all gross disease, thyroid bed, and bilateral levels II through VI lymph node levels were specifically targeted.

**Figure 1.**
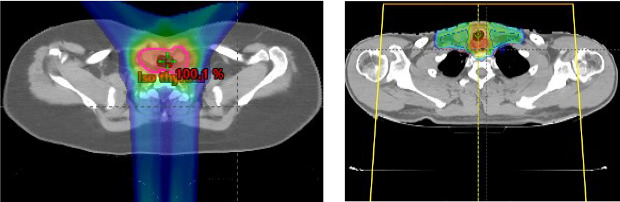
Planning protocols that used a combination of fields for adequate target coverage (left) or a single anterior field (right).

IMPT was delivered with a pencil-beam, active scanning delivery system using either single-field optimization IMPT or multifield optimization [24, 25]. Depending on the extent of tumor size and location, we developed planning protocols that used either a single anterior field or a posterior beam coupled with 2 anterior oblique beams for adequate target coverage (Figure 1).

In terms of the individual field simultaneous optimization technique, optimization target volumes of approximately 5 to 7 mm were generated as an expansion from the individual clinical target volumes in order to enhance effectiveness of robust optimization by accounting for patient-specific motion, setup-specific uncertainties, and proton relative stopping power–related range uncertainties. These independent errors were summed as quadrature (expressed empirically in the following equation, where CTV indicates clinical target volume).

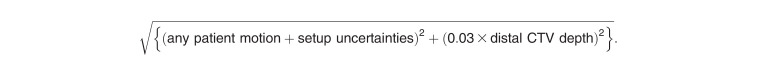



A scanning target volume was created to define a volume for IMPT spot placement by the optimizer. Dose painting of the low-, intermediate-, and high-risk clinical target volume is performed by cropping overlapping targets with a margin of 2 mm from each other to achieve homogeneous dose optimization. Motion management was not typically required based on 4-dimensional CT evaluation of respiratory and cardiac movement of the inferior mediastinum. However, repainting is often used based on approximately 5 mm of movement. In multifield IMPT, the system optimizes the weight of spots from multiple fields simultaneously to meet target coverage and normal tissue-sparing constraints. Other relevant treatment planning, immobilization, and image-guided RT details can be found in the referenced text [26].

## Statistical Analysis

Patient, tumor, and treatment characteristics were recorded and analyzed with descriptive statistics. Acute (during and 3 months after the last RT fraction) and late toxicities (>3 months after the last RT fraction) were graded and recorded according to the Common Terminology Criteria for Adverse Events version 5.0 (National Cancer Institute Bethesda, MD). Overall survival and local-regional recurrence-free survival were estimated using the Kaplan-Meier method. Overall survival was defined as the time elapsed from completing IMPT to death or last follow-up. Local-regional recurrence-free survival was defined as the time elapsed from completing IMPT to local-regional recurrence or last follow-up. Local-regional recurrence was defined as progression or recurrence of disease within the treatment volume. All analyses were performed using SAS version 9.4 (SAS Institute, Cary, North Carolina).

## Results

Median follow-up since completion of IMPT for all patients was 8 months (range, 3-40). Median age at treatment was 64 years (range, 40-77), and the majority were men (64%). All patients had an Eastern Clinical Oncology Group performance status ≤1. Histologies included papillary (55%), Hurthle cell (36%), and poorly differentiated (9%) carcinoma; 1 patient had tall cell variant. The majority of patients had biopsy-proven recurrent disease; 2 patients were diagnosed using PET/CT. Median number of resections before definitive IMPT was 2 (range, 1-4). Patient and tumor characteristics are presented in Table 1.

**Table 1.** Patient and tumor characteristics (N = 11).

**Patient**	**Age**	**Sex**	**Tobacco history**	**ECOG performance status**	**Histology**	**Diagnosis method**	**Recurrent**	**Prior RAI**	**Number of prior resections**	**Baseline Thyroglobulin**	**Post-treatment Thyroglobulin**
1	64	M	No	0	Papillary	PET/CT	Yes	2	2	38	3.5
2	64	F	No	0	Papillary	Biopsy	Yes	1	2	2.4	2.0
3	56	F	Yes	0	Hurthle cell	Biopsy	Yes	1	2	221	81
4	67	M	Yes	1	Hurthle cell	Biopsy	Yes	1	2	44	0.3
5	50	F	Yes	0	Papillary	Biopsy	Yes	6	4	255	111
6	55	M	No	0	Papillary tall cell variant	Biopsy	Yes	1	2	396	Not available
7	55	F	Yes	0	Hurthle cell	Biopsy	Yes	3	4	31	41
8	40	M	Yes	0	Papillary	Biopsy	Yes	1	2	1.4	0.9
9	77	M	Yes	1	Hurthle cell	PET/CT	Yes	1	2	112	21
10	67	M	No	1	Papillary	Biopsy	Yes	1	2	121	3.9
11	72	M	Yes	1	Poorly differentiated	Biopsy	Yes	1	3	101	1.2

**Abbreviations:** M, male; F, female; ECOG, Eastern Clinical Oncology Group; PET/CT, positron emission tomography/computed tomography; RAI, radioactive iodine.

IMPT was delivered with definitive intent. In the entire cohort, median RT dose was 70 Gy [RBE 1.1] (range, 60-70) delivered with a median of 33 fractions (range, 30-35). All patients received elective nodal irradiation. Concurrent chemotherapy was not administered for any patient in this cohort. Treatment characteristics are presented in Table 2.

**Table 2.** Treatment characteristics (N = 11).

**Patient**	**Intent**	**Total dose (Gy)**	**Fractions**	**Clinical target volumes**	**Concurrent chemotherapy**
1	Definitive	70	33	Local recurrence + bilateral neck LN	No
2	Definitive	70	35	Local recurrence + comprehensive left neck LN	No
3	Definitive	70	35	Local recurrence	No
4	Definitive	70	33	Local recurrence + bilateral neck LN	No
5	Definitive	66	30	Local recurrence + bilateral neck LN	No
6	Definitive	66	30	Local recurrence + bilateral neck LN	No
7	Definitive	60	30	Comprehensive left neck	No
8	Definitive	70	35	Comprehensive bilateral neck LN	No
9	Definitive	70	35	Local recurrence + bilateral neck LN	No
10	Adjuvant	66	33	Comprehensive bilateral neck LN	No
11	Adjuvant	66	30	Local recurrence + bilateral neck LN	No

**Abbreviation:** LN, lymph node.

**Figure 2.**
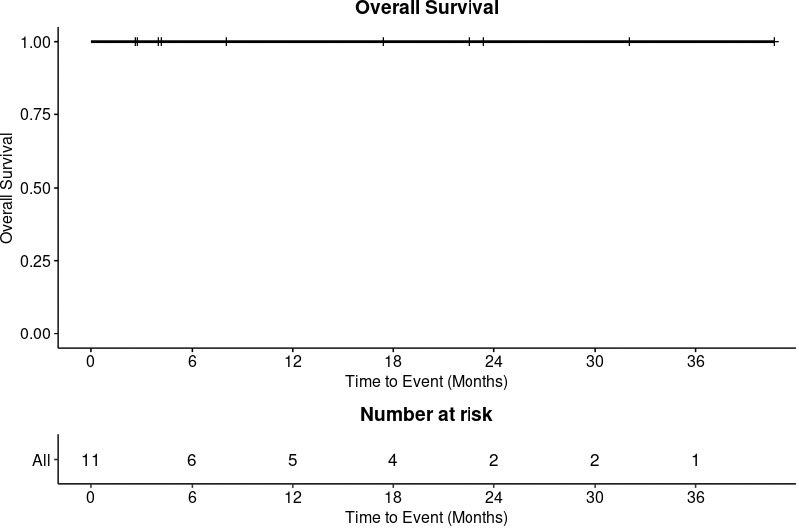
Overall survival.

**Figure 3.**
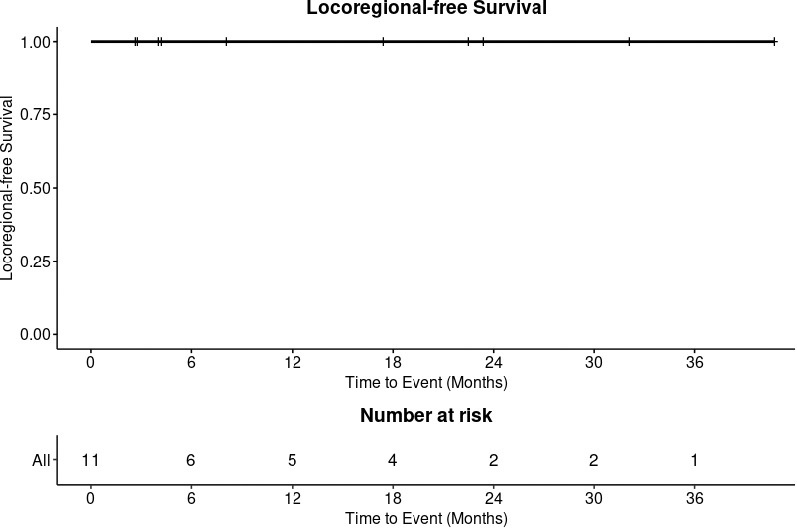
Local-regional–free survival.

At 8 months, all patients were alive (Figure 2) and without local-regional recurrence (Figure 3). Patient 11 experienced a delayed out-of-field nodal recurrence in the ipsilateral retropharyngeal space 4 months after completion of IMPT.

Three patients experienced acute treatment-related toxicities: grade 2 hoarseness, grade 2 xerostomia, and grade 3 weight loss requiring percutaneous endoscopic gastrostomy tube placement (each toxicity limited to 1 patient). During the course of RT, patient 12 required percutaneous endoscopic gastrostomy tube placement for severe weight loss secondary to thick secretions and dysphagia. The extent of disease in the patient who required percutaneous endoscopic gastrostomy tube placement required treating the right parotid gland (mean 42 Gy), left parotid gland (mean 35 Gy), pharyngeal constrictors (mean 58.5 Gy, V55Gy = 90%), cricopharyngeal inlet (mean 58.6 Gy), larynx (mean 54.5 Gy, V50Gy = 77.4%), cervical esophagus (mean 50.7 Gy, V35Gy = 100%), and esophagus (mean 42 Gy, V35Gy = 75.4%) above our typical constraints. The feeding tube was subsequently removed 4 months after completion of RT.

Four patients experienced late toxicities. Two patients with late grade 2 lymphedema of the neck were managed with physical therapy and supportive care. Two patients with late grade 3 esophageal stenosis required esophageal dilation procedures 9 months and 13 months after completion of RT. The first patient with late esophageal stenosis received a maximum dose of 72.7 Gy to the cervical esophagus and V55Gy = 43.1% due to extent of disease in the tracheoesophageal groove. The second patient with late esophageal stenosis also received prescription dose of 66 Gy to the cervical esophagus given the extent and location of disease. There were no grade 4 or 5 toxicities.

Seven patients completed patient-reported outcomes questionnaires at initiation and within 1 month of completing IMPT, including the European Organization for Research and Treatment of Cancer Quality of Life Questionnaire. Table 3 describes patient-reported outcomes related to dysphagia and hoarseness. There were no differences in pretreatment versus posttreatment patient-reported outcomes in terms of dysphagia or hoarseness.

**Table 3.** Patient-reported outcomes.

**Question**	**Pre-IMPT**	**Post-IMPT**	***P*****-value^1^**
Q5: Have you had problems swallowing liquids?			.22
Mean (SD)	1.4 (0.5)	1.9 (0.7)	
Median (range)	1.0 (1.0–2.0)	2.0 (1.0–3.0)	
Q6: Have you had problems swallowing pureed food?			.09
Mean (SD)	1.1 (0.4)	1.7 (0.8)	
Median (range)	1.0 (1.0–2.0)	1.0 (1.0–3.0)	
Q7: Have you had problems swallowing solid food?			.05
Mean (SD)	1.4 (0.5)	2.6 (1.3)	
Median (range)	1.0 (1.0–2.0)	3.0 (1.0–4.0)	
Q8: Have you choked when swallowing?			.32
Mean (SD)	1.4 (0.5)	1.7 (0.5)	
Median (range)	1.0 (1.0–2.0)	2.0 (1.0–2.0)	
Swallow score (transformed)			.08
Mean (SD)	11.9 (11.6)	32.1 (25.2)	
Median (range)	8.3 (0–33.33)	41.7 (0–66.7)	
Q16: Have you been hoarse?			.43
Mean (SD)	2.6 (0.9)	3.0 (1.0)	
Median (range)	3.0 (1.0–4.0)	3.0 (1.0–4.0)	

**Abbreviation:** IMPT, intensity-modulated proton therapy.

1 Wilcoxon signed-rank test with continuity correction.

## Discussion

The role of EBRT in differentiated thyroid cancer remains controversial. There is no prospective clinical trial data for treatment of recurrent, RAI-refractory DTC, a particularly poor prognostic subgroup of patients in which EBRT may be a valuable treatment option for improving local-regional control. Our early experience demonstrated that PBT is a viable treatment option with a promising local-regional control rate for patients with recurrent, RAI-refractory DTC.

In our study, all patients with recurrent, RAI-refractory DTC who received definitive IMPT were alive at last follow-up. Additionally, IMPT demonstrated promising local-regional recurrence-free survival rate of 100% at last follow-up. A contemporary experience from Memorial Sloan-Kettering Cancer Center of definitive photon-based EBRT with or without concurrent chemotherapy for gross residual or unresectable non-anaplastic and non-medullary thyroid cancer reported a 3-year local-regional progression-free survival rate of 77.3% for 66 patients, 77.3% of whom were treated with IMRT [27]. Concurrent chemotherapy was associated with a higher rate of grade-3 hoarseness (10% vs 0%, *P* = .033) and a nonsignificant improvement in progression-free survival (90% vs 73%, *P* = .347). Our series demonstrated that PBT might offer further improvement in local-regional control compared with modern experiences with photon therapy, particularly for recurrent, RAI-refractory DTC. Further evidence from a Hong Kong study supports the role of EBRT in patients with gross residual disease after thyroidectomy, as the addition of EBRT improved local control (56 vs 24%) [6]. A similar group of patients with incompletely resected disease demonstrated a potential survival benefit at 5 years with the addition of EBRT (77 vs 37%) [7]. Studies have reported a potential benefit of EBRT in patients with microscopic residual disease, especially in older patients with advanced disease [8–15]. A pooled analysis demonstrated that regardless of stage or presence of residual disease, the recurrence rate in patients who received EBRT was 8% versus 25% in those who did not receive EBRT [8]. Tam et al [12] reported that in a cohort of 88 patients with T4a DTC, the addition of EBRT to radioiodine improved 5-year disease-free survival rate to 57% versus 43%. Ultimately, maximizing local-regional cancer control of DTC is vital to avoid cancer-related morbidity and mortality. Given the promising local-regional control rate in our series, IMPT may be considered a viable treatment option in the definitive setting.

The clinical decision to use EBRT can be difficult because head and neck RT is associated with significant risk of toxicity. Recurrences after surgery are frequently located in the tracheoesophageal groove, and, as a result, patients are facing extensive surgery such as total laryngopharyngectomy, partial esophagectomy, and tracheal resection as salvage treatment. Treating this location with a definitive RT dose will have expected laryngeal and esophageal toxicities as seen in our study. Additionally, the recurrent, RAI-refractory DTC patients in our cohort were at high risk of toxicity as they had a median of 2 prior surgeries, excluding ethanol ablations and RAI treatments before receipt of EBRT. Clinically severe acute toxicities were limited to 1 patient (8%) with dysphagia, requiring temporary feeding tube placement. In the Memorial Sloan Kettering experience of EBRT for non-anaplastic nonmedullary thyroid cancer, 23% of patients required a gastrostomy tube before initiation of EBRT, and 12% of patients required a gastrostomy tube during or within 90 days of completing EBRT [27]. The dosimetric advantages of IMPT compared with conventional photon techniques may allow for improved sparing of the pharyngeal constrictor muscles, cricopharyngeal inlet, and larynx, potentially diminishing the risk of feeding tube placement during or after RT. Grade 3 late toxicities in our cohort included 2 cases of esophageal strictures that required esophageal dilation about 1 year after treatment. Both patients received prescription dose to the cervical esophagus due to target proximity to the organ at risk, which was largely unavoidable. The relative biological effectiveness, typically calculated using a constant of 1.1 relative to photon therapy, can vary significantly in different regions of the proton beam. Regions of high linear energy transfer may have accounted for observed toxicity given that the normal tissue received a dose that was within typical constraints ]28–30]. The consequence of the relative biological effectiveness calculated as a function of dose, linear energy transfer, and radiosensitivity of the normal tissue is currently an area of study including methods to redistribute areas of high linear energy transfer away from critical organs at risk [31]. Further investigation is needed to understand and prevent esophageal and laryngeal toxicity after PBT. Importantly, there were no differences in pretreatment versus posttreatment patient-reported outcomes in terms of dysphagia or hoarseness. No patients in our study experienced grade 4 or 5 toxicities.

This study has several limitations. We acknowledge that this is a small retrospective series of patients with a short period of follow-up. Therefore, it is difficult to make conclusions about the long-term safety and efficacy of IMPT for this subgroup of patients. However, this is a detailed early-outcomes report of patients treated at a high-volume academic, tertiary cancer center. Our findings suggest that IMPT is a definitive treatment option with promising local-regional control for patients with recurrent, RAI-refractory DTC. Further study with longer follow-up is needed to better understand long-term clinical outcomes for this high-risk patient population.

## Conclusion

Our early experience suggests that IMPT is a viable treatment option for recurrent, RAI-refractory DTC with promising local-regional control. Further study is warranted to evaluate the long-term efficacy and safety of IMPT in this patient population.
